# Assessing the Role of Intrathecal Catheters in Preventing Post-dural Puncture Headaches: A Systematic Review of Current Evidence

**DOI:** 10.7759/cureus.100456

**Published:** 2025-12-30

**Authors:** Isha Chopra, Abdullah Kilic, Ayushi Saxena, Bilal Khan, Rupanshu Rupanshu, Russaal S Mann, Iana A Malasevskaia

**Affiliations:** 1 Anesthesia, Government Medical College, Dausa, Mitrapura, IND; 2 Internal Medicine, Hackensack University Medical Center, Hackensack, USA; 3 Medicine, California Institute of Behavioral Neurosciences and Psychology, Fairfield, USA; 4 Health Sciences, Queen's University, Kingston, CAN; 5 Internal Medicine, St. Martinus University Faculty of Medicine, Willemstad, CUW; 6 Internal Medicine, Vardhman Mahavir Medical College and Safdarjung Hospital, New Delhi, IND; 7 Hospital-Based Medicine, Harvard T.H. Chan School of Public Health, Boston, USA

**Keywords:** accidental dural puncture, epidural blood patch, intrathecal catheters, neuraxial complications, post-dural puncture headache, systematic review

## Abstract

Post-dural puncture headache (PDPH) is defined as a postural headache that frequently develops within five to seven days following dural puncture, typically due to persistent cerebrospinal fluid (CSF) leaks. Young individuals and the obstetric population are particularly affected, and if not managed timely and appropriately, PDPH can lead to serious repercussions. This systematic review aims to assess the efficacy of intrathecal catheter placement in reducing the incidence of PDPH following accidental dural puncture (ADP).

Following the Preferred Reporting Items for Systematic Reviews and Meta-Analyses (PRISMA) guidelines, a comprehensive search across multiple databases identified 11 relevant studies, including randomized controlled trials (RCTs), cohort studies, case series, and case reports, with sample sizes ranging from one to 1001. Inclusion criteria focused on adults (≥18 years) who experienced ADP, comparing those receiving intrathecal catheter placement to those who did not. Quality assessment tools were employed to evaluate the methodological rigor of the included studies.

The synthesized evidence suggests that intrathecal catheter placement may be associated with lower rates of PDPH compared to standard management in many studies, and it appears to have a favorable safety profile. However, significant heterogeneity in study designs, populations, and outcome definitions precludes a definitive conclusion.

Intrathecal catheter placement appears to be a promising intervention for managing ADP, potentially reducing PDPH incidence. Given the variability in evidence, further rigorous research, particularly large-scale RCTs, is required to confirm efficacy and establish standardized protocols for clinical use.

## Introduction and background

Accidental dural puncture (ADP) during epidural anesthesia or analgesia can lead to post-dural puncture headache (PDPH), a common complication that significantly affects patient recovery [1]. PDPH may develop because of various factors, including multiple attempts to site an epidural or the use of wider gauge needles [2]. Studies stipulate that almost more than 50% of patients experiencing ADP develop PDPH, with some reports suggesting an incidence as high as 76-85% [1,3].

The incidence of PDPH is notably higher among specific populations, particularly young females and obstetric patients [4]. This condition can be debilitating, hindering mobility and delaying recovery after surgery or delivery, thereby contributing to significant distress and morbidity, especially among parturients [5]. A closed claims analysis by the American Society of Anesthesiologists in 2003 found that headaches accounted for approximately 12% of lawsuits against anesthetists, underscoring the need for increased vigilance in obstetric anesthesia practice [6]. If left untreated, PDPH can lead to severe complications, including chronic headache, subdural hematoma, reversible cerebral vasoconstriction syndrome, or cortical thrombosis [7]. Notably, Webb et al. reported a 28% incidence of chronic headache in women with ADP using a 17G epidural needle [8].

Effective management of PDPH requires not only reassurance but also a mechanism-directed treatment approach, coupled with stringent follow-up. Over the years, treatment strategies have evolved, ranging from pharmacological to non-pharmacological interventions. These include bed rest, oral hydration, caffeine, adrenocorticotropic hormone, epidural morphine, gabapentin, and various interventional techniques [9]. Recent literature has also explored the efficacy of nebulized dexmedetomidine, intravenous neostigmine, dexamethasone, and nerve blocks; however, the evidence supporting these interventions remains insufficient [10].

One technique that has garnered attention is the placement of an intrathecal catheter (ITC) following ADP, intended to prevent PDPH, when kept for a duration of 24 hours. However, recent evidence regarding the effectiveness of ITCs is equivocal [11]. In light of these considerations, we undertook this systematic literature review to evaluate the efficacy of ITC insertion following ADP as a strategy for mitigating the risk of PDPH and reducing the incidence of epidural blood patch (EBP) procedures.

The anticipated benefits of this intervention are multifaceted. Primarily, it may lead to a significant reduction in both the severity and duration of PDPH, thereby enhancing patient comfort and overall satisfaction during the recovery process. Furthermore, effective management of PDPH through this approach could potentially decrease healthcare costs associated with extended hospital stays and the need for additional treatments. Ultimately, this could contribute to an improvement in the quality of care provided to patients experiencing ADP.

## Review

Methods

This systematic review was conducted in accordance with the Preferred Reporting Items for Systematic Reviews and Meta-Analyses (PRISMA) 2020 guidelines [12]. The review aimed to evaluate the effect of ITC placement on the incidence of PDPH following an ADP during epidural procedures. The primary research question guiding this review was the following: What is the effect of ITC placement on the incidence of PDPH after an ADP?

Eligibility Criteria

Eligibility criteria for this review were established to ensure the inclusion of relevant studies. Inclusion criteria encompassed original research studies, including randomized controlled trials (RCTs), observational studies, as well as case reports and case series. The population of interest consisted of adults aged 18 years and older who had experienced an ADP during the placement of an epidural catheter for anesthesia or analgesia. Studies assessing the placement of an ITC following an ADP were included. Furthermore, only studies reporting the incidence of PDPH as an outcome were considered. The review included studies published in English without a specific publication time frame.

Exclusion criteria were also defined to filter out studies that did not meet the necessary standards. Non-original research, including reviews, meta-analyses, abstracts, commentaries, and editorials, preprints, and studies without full data or unpublished results also were excluded. Additionally, studies involving pediatric patients or those with pre-existing conditions affecting headache incidence, such as chronic migraine, were not considered. Studies that did not specifically assess the placement of an ITC following an ADP or lacked clear definitions or measurements of PDPH were also excluded.

Literature Search

The literature search was conducted across multiple databases, including PubMed/MEDLINE, ScienceDirect, ClinicalTrials.gov, Europe PMC, and Cochrane Library. The search strategy was developed using key concepts relevant to the research question. The search included terms such as "intrathecal catheter", "accidental dural puncture", and "dural puncture headache". The search was initiated on November 25, 2024, and completed on December 6, 2024. Filters applied during the search included full text, case reports, clinical studies, clinical trials, and observational studies, all published in English (Table 1).

**Table 1:** Literature search

Search strategy	Database/register	Number of studies (before filters)	Filters	Date of search	Number of studies (after filters)
“Accidental dural puncture” Or “unintentional dural Puncture” / OR “Dural tap complication” And Post Dural Puncture headache/ Complication “[Majr] OR “Post-Dural puncture headache/ prevention and control”[Majr] OR “Post-Dural Puncture Headache/therapy” [majr] ) AND “Intrathecal catheter” OR “subarachnoid catheter”	PubMed/MEDLINE	37	Free full text, clinical studies, clinical trials, observational studies, English language	25.11.2024	14
(“accidental dural puncture OR Unintentional dural puncture AND (“Intrathecal catheter OR Subarachnoid catheter”) AND (‘’ post dural puncture headache ”)	ScienceDirect	499	Research articles, articles in English language only, open access and open archive	31.11.24	31
Condition/ disease: (“dural puncture headache” OR “post dural puncture headache”) Intervention/ treatment: (“Intrathecal catheter” OR “subarachnoid catheter ”)	ClinicalTrials.gov	5	Completed studies, 18-65 years of age, interventional and observational studies, studies with results	31.11.24	2
accidental dural puncture AND (Intrathecal catheter OR Subarachnoid catheter) AND post dural dural puncture”) 874	Europe PMC	202	Full text, research articles	31.11.2024	142
Search Hits #1 ("intrathecal catheter" OR "intrathecal injection" OR "spinal catheter" ) 834 #2 (“accidental dural puncture” OR “ dural puncture”) 874 #3 ("dural puncture headache " OR "post- dural puncture headache " OR "spinal headache") 597 #4 MeSH descriptor: [Post- Dural Puncture Headache ] explode all trees	Cochrane Library	28	Full text, English	6.12.2024	13

Screening of the Included Studies

Upon completing the initial search and gathering the final selection of studies, the chosen articles were imported into the Rayyan application (Rayyan Systems Inc., Cambridge, Massachusetts, United States) for further evaluation [13]. This tool aided in organizing and managing the studies, promoting effective collaboration among the reviewers. The screening was carried out by two independent reviewers (I.C. and A.K.) who examined the titles and abstracts of the identified articles to determine their eligibility. Any disagreements that arose were resolved through discussion, and if needed, a third author (I.A.M.), acting as a mentor, was consulted to achieve a consensus. After the preliminary screening, the full texts of the potentially relevant studies were scrutinized to verify eligibility according to the predetermined criteria.

Data Collection Process

A standardized, pre-piloted data extraction form was used. Two independent reviewers extracted data from the included studies. Any discrepancies were resolved through discussion or by consultation with a third reviewer. The extracted data included study design, population characteristics, sample size, details of the intervention (catheter type, duration), comparator, primary outcomes (PDPH incidence, EBP requirement), and reported adverse events.

Data Items

The primary data items extracted for synthesis were as follows: (1) incidence of PDPH, (2) rate of EBP administration, and (3) nature and frequency of adverse events related to the intervention. Secondary items included study country, population specifics (e.g., obstetric vs. non-obstetric), and technical details of ITC management.

*Quality Appraisal* 

Quality appraisal was performed independently by two authors. Any discrepancies were resolved through discussion, and if there was a lack of agreement, a third author was consulted.

The quality of the included studies was assessed using appropriate tools, including the Joanna Briggs Institute (JBI) Checklist for Case Reports [14], the JBI Checklist for Case Series [15], the Cochrane Risk of Bias 2 (RoB 2) tool for RCTs [16], and the Newcastle-Ottawa Scale (NOS) for observational studies [17]. 

Synthesis Methods

Due to significant clinical and methodological heterogeneity among the included studies (variations in design, population, intervention protocols, and outcome definitions), a meta-analysis was not deemed appropriate. Therefore, a narrative synthesis was conducted. Findings were grouped and summarized thematically (e.g., by outcome: PDPH incidence, EBP rate) and by study design (RCTs, cohort studies) to explore patterns and inconsistencies in the evidence. This qualitative synthesis is presented in the Results section.

Results

**Figure 1:**
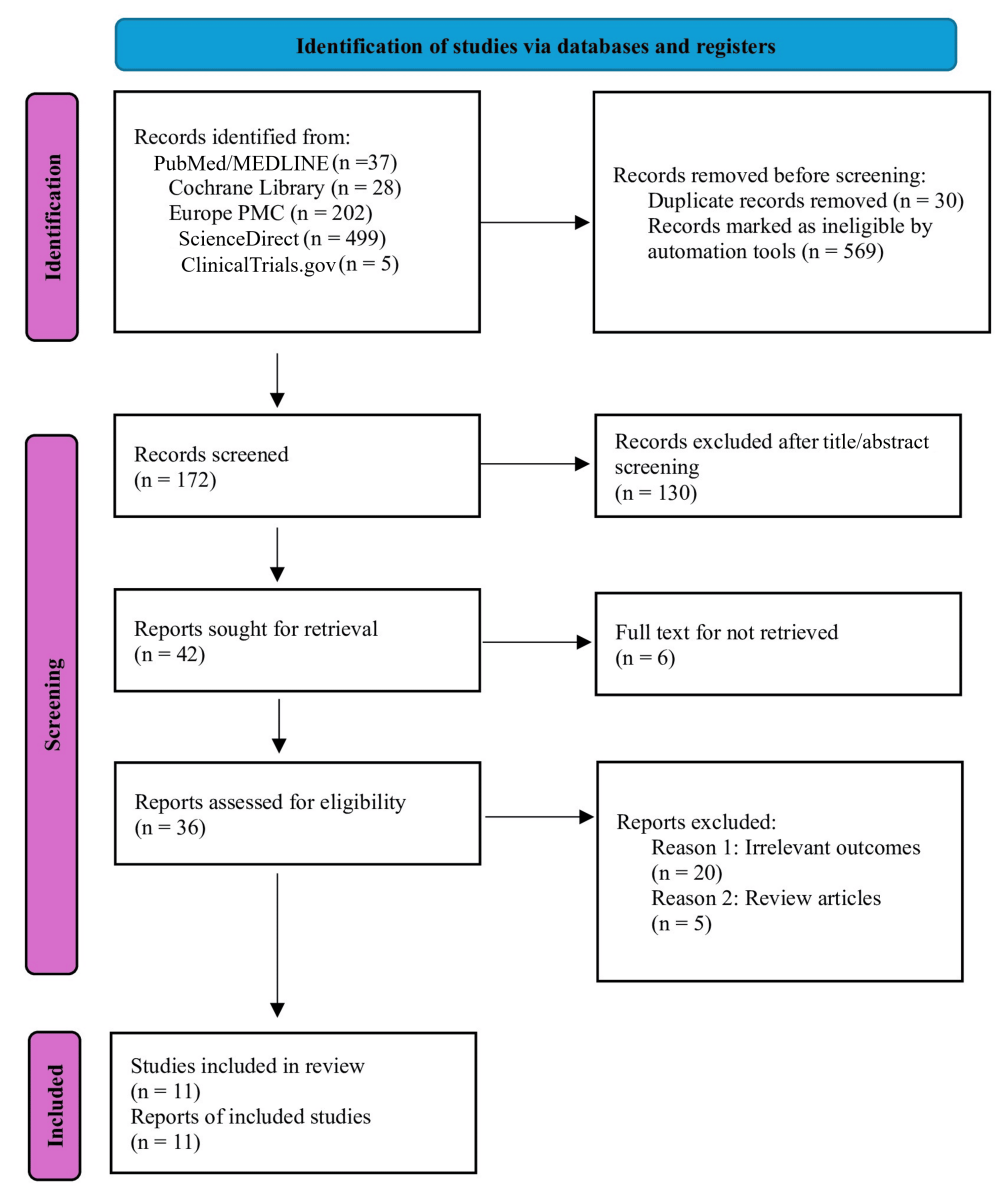
Detailed PRISMA 2020 flow diagram PRISMA: Preferred Reporting Items for Systematic Reviews and Meta-Analyses

Following the PRISMA 2020 guidelines [12], our search strategy identified a total of 771 studies. After applying filters and removing duplicates, 172 records were screened, which led to 36 records being assessed for eligibility. Ultimately, 11 studies were included for quality appraisal and analysis, as illustrated in the PRISMA 2020 flow diagram (Figure 1).

Quality Appraisal

Quality appraisal was performed independently by two authors using appropriate tools based on the study design. Table 2 presents the quality of the cohort studies assessed using the NOS [17]. The majority of these studies scored well, indicating a high level of methodological rigor and reliability in their findings.

**Table 2:** Quality appraisal of cohort studies using the Newcastle-Ottawa Scale

Author/year	Study design	Selection	Comparability	Outcome/exposure	Total score
Cohen et al., 1994 [18]	Retrospective cohort	4	2	3	9
Ayad et al., 2003 [19]	Retrospective cohort	4	1	3	8
Jadon et al., 2009 [20]	Prospective cohort	3	1	3	7
Gupta et al., 2020 [21]	Prospective cohort	3	2	3	8
Creazzola et al., 2023 [22]	Retrospective cohort	4	2	3	9
Kaddoum et al., 2014 [23]	Retrospective cohort	4	1	3	8

Table 3 summarizes the risk of bias for the RCTs evaluated using the Cochrane RoB 2 tool [16]. The overall assessment indicates that while both studies generally exhibited low risk in several domains, some concerns were noted, particularly regarding deviations from the intended interventions and selection of reported results.

**Table 3:** Quality appraisal of randomized clinical trials using the Cochrane Risk of Bias 2 tool D: domains; D1: bias due to the randomization process; D2: deviation from intended intervention; D3: missing outcome data; D4: measurement of outcomes; D5: selection of the reported result

Author/year	D1	D2	D3	D4	D5	Overall
Russell 2012 [24]	Low risk	Some concerns	Low risk	Low risk	Some concerns	Some concerns
Arkoosh et al., 2008 [25]	Low risk	Some concerns	Low risk	Low risk	Low risk	Some concerns

Table 4 presents the quality appraisal of the case series conducted by Chaudhary et al. [26], using the JBI quality appraisal checklist. The assessment revealed that the study met eight out of the 10 criteria, indicating a generally good quality, although some concerns were noted regarding the consecutive inclusion of participants and the reporting of demographics.

**Table 4:** Quality appraisal of case series using the Joanna Briggs Institute quality appraisal checklist Q: question

Joanna Briggs Institute quality appraisal checklist		
Q1	Were there clear criteria for inclusion in the case series?	Yes
Q2	Was the condition measured in a standard, reliable way for all participants included in the case series?	Yes
Q3	Were valid methods used for the identification of the condition for all participants included in the case series?	Yes
Q4	Did the case series have consecutive inclusion of participants?	No
Q5	Did the case series have complete inclusion of participants?	Yes
Q6	Was there clear reporting of the demographics of the participants in the study?	No
Q7	Was there clear reporting of clinical information of the participants?	Yes
Q8	Were the outcomes or follow-up results of cases clearly reported?	Yes
Q9	Was there clear reporting of the presenting site(s)/clinic(s) demographic information?	Yes
Q10	Was statistical analysis appropriate?	Yes
	Overall appraisal/decision	8/10 included

Additionally, Table 5 summarizes the quality appraisal of case reports by Emyedu et al. [27] and Matturu et al. [28], also using the JBI quality appraisal checklist. Both reports demonstrated strong quality, meeting seven and six, respectively, out of the eight criteria, indicating that the essential elements of case reporting were well addressed.

**Table 5:** Quality appraisal of case reports using the Joanna Briggs Institute quality appraisal checklist Q1. Were the patient's demographic characteristics clearly described? Q2. Was the patient's history clearly described and presented as a timeline? Q3. Was the current clinical condition of the patient on presentation clearly described? Q4. Were diagnostic tests or assessment methods and the results clearly described? Q5. Was the intervention(s) or treatment procedure(s) clearly described? Q6. Was the post-intervention clinical condition clearly described? Q7. Were adverse events (harms) or unanticipated events identified and described? Q8. Does the case report provide takeaway lessons? Q: question

Author/year	Q1	Q2	Q3	Q4	Q5	Q6	Q7	Q8	Overall appraisal/decision
Emyedu et al., 2021 [27]	Yes	No	Yes	Yes	Yes	Yes	Yes	Yes	7/8 included
Matturu et al., 2022 [28]	Yes	No	Yes	Yes	Yes	Yes	No	Yes	6/8 included

Study Design Overview

In our review, a total of 11 studies were included, of which six are cohort studies, comprising both retrospective and prospective designs. Furthermore, the review encompasses two RCTs, one case series, and two case reports. This diverse array of study designs contributes to the robustness of the findings related to interventional management of PDPH in patients. The sample sizes across these studies vary widely, ranging from a single participant to 1001, thereby providing a comprehensive assessment of the efficacy, safety, and overall impact of various treatment modalities for PDPH (Table 6).

**Table 6:** Characteristics of the included studies CSA: continuous spinal analgesia; CSE: combined spinal epidural; ASA: American Society of Anesthesiologists; CIT: continuous intrathecal; CEPI: continuous epidural infusion; EBP: epidural blood patch; PDPH: post-dural puncture headache; ITC: intrathecal catheter; NRS: Numerical Rating Scale

Study reference	Study design/sample size/country	Population	Intervention/comparison	Key findings	Adverse effects
Cohen et al., 1994 [18]	Retrospective cohort study. Sample size: 45 patients. Conducted in the USA (Weiler Hospital, Albert Einstein College of Medicine)	Obstetric patients who experienced accidental dural puncture during epidural block attempts for caesarean delivery	Intervention: CIT analgesia with fentanyl, bupivacaine, and epinephrine (Group III). Comparison: Group I (standard epidural), Group II (continuous spinal for the duration of surgery)	No patients in Group III developed PDPH. PDPH incidence: 33% in Group I, 47% in Group II (significant difference; p<0.009). Excellent pain relief in Group III; patients ambulated easily without significant side effects	No reports of sensory loss, weakness, nausea, vomiting, pruritus, or respiratory issues in Group III. No neurological symptoms or signs of infection reported
Ayad et al., 2003 [19]	Retrospective cohort study. Sample size: 115 patients. Conducted in Cleveland, OH (Fairview Hospital, Cleveland Clinic Health System)	Obstetric patients who experienced accidental dural puncture during epidural block attempts for caesarean delivery	Intervention: Group I CEPI with bupivacaine and sufentanil, catheter removed after delivery; Group II CITI and catheter removed after delivery; Group III CIT analgesia and catheter left in place for 24 hours after delivery	PDPH incidence: 91% in Group I, 51.4% in Group II, and 6.2% in Group III. Incidence of EBP: 81.1% in Group I, 31.4% in Group II, and 3.1% in Group III	No neurological sequelae or signs of infection reported
Jadon et al., 2009 [20]	Prospective cohort, 34 patients, India	Heterogeneous adult patients who developed accidental dural puncture during CSE	20G epidural catheter placed intrathecally and left for 24-36 hours	Incidence of PDPH: 11.76% , with two patients needing EBP	Transient paresthesia during ITC insertion in two patients
Gupta et al., 2020 [21]	Prospective, multicenter, international, cohort study; 1001 patients from 24 countries	Obstetric patients who developed PDPH after accidental dural puncture	EBP vs. no EBP 647 (64.6%) receiving an EBP and 354 (35.4%) not receiving EBP	Headache intensity: higher in the EBP group (mean NRS 8.0) vs. the no-EBP group (mean NRS 6.9). Spontaneous recovery: 5.8% within 24 hours; 12.2% in the no-EBP group vs. 2.2% in the EBP group. Second EBP required: 19.6% of EBP patients; higher if EBP performed <24 hours after PDPH diagnosis (24% vs. 15%). Minimal orthostatic component: 6.4% of patients; 8.8% in the no-EBP group vs. 5.1% in the EBP group	Intracranial bleeding: 3 patients (0.46%) in the EBP group. Chronic headache: 5% reported at 3 months (6.9% EBP vs. 1.7% no-EBP). Persistent backache: 14% reported at 3 months (17% EBP vs. 8.8% no-EBP). Other symptoms: neck stiffness, auditory/visual symptoms, nausea. Medication use: 10.1% on medication for headache/backache at 3 months
Creazzola et al., 2023 [22]	Retrospective cohort study. Sample size: 60 patients. Conducted in Rome, Italy (Obstetric Unit of the San Giovanni Calibita Hospital)	Obstetric patients who experienced accidental dural puncture during epidural block attempts for caesarean delivery	Intervention: CIT analgesia with infusion pumps for 36 hours (Group I). Comparison: standard epidural catheter in the same or different space	Headache incidence: 60.5% in Group I, 84.6% in Group II (p=0.107). No patient in either group required EBP	No adverse events reported
Kaddoum et al., 2014 [23]	Retrospective cohort study. Sample size: 23. Conducted in the USA (Hutzel Women's Hospital, Detroit)	Women who experienced accidental dural puncture during attempted labor epidural	Intervention: ITC group: inserting the epidural catheter intrathecally (54 patients); non-ITC group: repeat epidural block (184 patients)	Incidence of PDPH: 54% in the non-ITC group vs. 37% in the ITC group. Incidence of EBP: 31% in the non-ITC group vs. 13% in the ITC group	No adverse effects reported
Russell 2012 [24]	Prospective controlled study, 115 women recruited; 97 compliant with the protocol (47 in the repeat epidural group, 50 in the spinal group) (UK)	Women who experienced accidental dural puncture during attempted labor epidural analgesia	Group A: repeat epidural analgesia. Group B: CSA (inserting the epidural catheter intrathecally)	No significant difference in PDPH rates: spinal 72% vs. epidural 62% (p=0.2). EBP rates: spinal 50% vs. epidural 55% (p=0.6). Higher attempts for neuraxial analgesia in the repeat epidural group (41% vs. 12%; p=0.0004)	Over one-third experienced complications, three times higher than the spinal group. Specific complications: difficulty threading catheters. Need for two or more attempts to achieve neuraxial analgesia: 41% (repeat epidural) vs. 12% (spinal) (p=0.0004). Risk of second dural puncture: 9% of women in the repeat epidural group experienced a second dural puncture
Arkoosh et al., 2008 [25]	Randomized, double-masked, multicenter trial; 325 women in the CIT group and 100 in the CEPI group (7 hospitals) (USA)	ASA I/II parturients aged 18-45 in spontaneous or induced labor with healthy, vertex fetuses at term	Intervention: CIT using a 28G catheter. Comparison: CEP using a 20G catheter	98% comfort after initial bolus in CIT vs. 93% in CEPI. Adequate analgesia: 88.4% (CIT) vs. 92% (CEPI). Lower visual analog scores in CIT for 60 minutes post-injection. No significant difference in nausea or headache, but higher pruritus in CIT. Higher Bromage scores (less blockade) in CIT (4.9% <4) vs. CEPI (13%). Similar satisfaction rates postpartum (88.9% CIT, 93.1% CEPI)	Slightly more headaches and significantly higher incidence of pruritus in CIT. Technical issues: 3.7% failure in CIT vs. 3% in CEPI for catheter placement. Difficult catheter removal in CIT (8.1% rated difficult) compared to CEPI (0%). No permanent neurologic deficits reported; <1% chance of deficits with 28G catheter
Chaudhary et al., 2014 [26]	Case series on CSA after accidental dural puncture. 11 patients. Tertiary care hospital, India	Adult patients for pelvic, non-obstetric surgery under CSE	CSA using an epidural catheter after accidental dural puncture	Successful spinal anesthesia, intrathecal morphine for postoperative pain relief. No need for rescue analgesics	No reports of prolonged motor blockade, hemodynamic instability, desaturation or need for rescue analgesics
Emyedu et al., 2021 [27]	Case report on CSA after accidental dural puncture. 1 patient (38-year-old man). University hospital, Uganda	ASA physical status 1E; 38-year-old man with abdominal pain and intestinal obstruction	CSA using an epidural catheter after accidental dural puncture	Successful use of CSA for emergency exploratory laparotomy. Pain scores: 0/10 at 12 hours and 2/10 at 24 hours and 72 hours. Discharged on postoperative day 3 with no neurologic sequelae	Episode of hypotension managed with fluids and infusion adrenaline
Matturu et al., 2022 [28]	Case report on CSA after accidental dural puncture. 1 patient (49-year-old woman). Rural hospital, USA	ASA physical status 1E; 49-year-old woman with abdominal discomfort and intestinal blockage	CSA using an epidural catheter after accidental dural puncture	Successful use of CSA for emergency exploratory laparotomy. Pain scores: 0/10 at 12 hours, 2/10 at 24 hours, and 2/10 at 72 hours. Discharged on postoperative day 15 with no neurologic side effects	Episode of hypotension during procedure (managed with fluids). No permanent neurologic side effects reported

Synthesis of Results

Across the six cohort studies, five [18-20,22,23] reported a lower incidence of PDPH in groups where an ITC was placed compared to control groups (standard epidural or repeat epidural). The magnitude of reduction varied widely, from 0% vs. 33% to 37% vs. 54% [18,23]. In contrast, the RCT by Russell found no significant difference in PDPH rates between spinal (72%) and repeat epidural (62%) groups [24]. A similar trend was observed for the requirement of therapeutic EBP. Studies by Ayad et al. and Kaddoum et al. reported substantially lower EBP rates in their ITC groups (3.1% and 13%, respectively) compared to controls (81.1% and 31%) [19,23]. The safety profile was generally favorable. Commonly reported issues were transient and manageable, such as procedural paresthesia or hypotension [27,28]. No studies reported serious neurological sequelae or infections directly attributable to the ITC.

Discussion

Summary of Main Findings

This systematic review synthesizes evidence on the effect of ITC placement on the incidence of PDPH following ADP. The collated findings from RCTs, cohort studies, and case reports suggest a potential benefit, with several studies reporting lower PDPH and EBP rates in groups receiving an ITC [18-23,26-28]. However, this evidence is characterized by significant heterogeneity in study design, patient populations, procedural protocols (e.g., catheter gauge, duration), and outcome definitions. While the safety profile appears favorable, the lack of consistent, high-quality evidence precludes a definitive conclusion about efficacy, highlighting a critical gap between promising clinical observations and robust proof.

This systematic review examines the effect of ITC placement on the incidence of PDPH following ADP. PDPH is a common complication that can significantly impact patient recovery and quality of life. By synthesizing evidence from various study designs, including RCTs, cohort studies, case series, and case reports, we aim to provide a comprehensive understanding of the effectiveness of this intervention.

Evidence from RCTs

The clinical trials conducted by Russell and Arkoosh et al. offer contrasting perspectives on managing PDPH [24,25]. Russell's prospective controlled study involved 115 women in the United Kingdom who experienced ADP during labor epidural analgesia [24]. The study compared repeat epidural analgesia (Group A) with continuous spinal analgesia (Group B). Although both groups exhibited high rates of PDPH (72% in spinal vs. 62% in epidural), the repeat epidural group faced a significantly higher incidence of complications, including difficulty in catheter threading and a notable risk of second dural puncture (9%) [24]. This raises concerns about the safety of repeat epidural procedures in this population.

In contrast, Arkoosh et al.'s randomized, double-masked trial included 425 women across seven hospitals in the United States and focused on comparing continuous intrathecal (CIT) with continuous epidural (CEPI) [25]. The results demonstrated a higher comfort level in the CIT group, with 98% reporting comfort after the initial bolus compared to 93% in the CEPI group. Although pruritus was more prevalent in the CIT group, no permanent neurologic deficits were reported [25]. This study suggests that CIT may provide superior pain management and comfort, particularly in obstetric settings.

Evidence from Cohort Studies

Cohort studies provide the most substantial evidence for a potential benefit of ITC placement, though inherent design limitations warrant cautious interpretation. The findings consistently demonstrate lower PDPH incidence with ITC compared to alternative management. Cohen et al. reported a 0% PDPH rate in the CIT group versus 33% in a standard epidural group (p<0.009) [18]. Ayad et al. found a dramatic reduction: PDPH incidence was 91% in the standard group, 51.4% with immediate catheter removal, and 6.2% when the catheter was left for 24 hours [19].

Later studies corroborated this trend. Jadon et al. reported an 11.76% PDPH incidence following ITC placement [20]. Kaddoum et al. observed rates of 37% (ITC) versus 54% (repeat epidural), with corresponding EBP rates of 13% vs. 31% [23]. Creazzola et al. noted a lower headache incidence with CIT (60.5%) versus standard epidural (84.6%), though this did not reach statistical significance (p=0.107) [22].

The large international cohort by Gupta et al. added nuance, comparing EBP to conservative management. While not a direct ITC study, it revealed higher headache intensity (mean NRS 8.0 vs. 6.9) and a 19.6% rate of second EBP in the intervention group, highlighting variability in procedural outcomes [21].

The safety profile across these studies was favorable. No serious neurological sequelae or infections were attributed to ITC placement, with only transient paresthesia reported occasionally [18-20,23]. Collectively, these observational data suggest ITC placement is associated with reduced PDPH risk and is well-tolerated, though confirmation from higher-level evidence is required.

Evidence from Case Series and Case Reports

Case evidence, while representing a low level on the efficacy hierarchy, demonstrates the successful clinical application and safety of continuous spinal analgesia following ADP. Chaudhary et al. reported a series of 11 non-obstetric surgical patients where continuous spinal analgesia provided effective anesthesia and analgesia without rescue medication or major complications [26]. Individual reports further illustrate its utility in emergencies. Emyedu et al. documented its use in a 38-year-old man undergoing exploratory laparotomy, with excellent pain scores (0/10 at 12 hours, 2/10 at 24-72 hours) and discharge on postoperative day 3, despite manageable intraoperative hypotension [27]. Matturu et al. reported a similar outcome in a 49-year-old woman, with comparable pain control and discharge on day 15 [28]. These reports confirm the technical feasibility and favorable immediate safety profile of continuous spinal analgesia for providing analgesia after ADP, though they do not establish its efficacy for PDPH prevention.

Mechanisms and Clinical Heterogeneity

The variability in outcomes across studies can be attributed to several key factors. First, technical heterogeneity is substantial: studies differed in catheter gauge (e.g., 20G vs. 26G), duration of placement (immediate removal vs. 24-36 hours), and drug infusion protocols [18,20,25]. Second, population differences existed, with most evidence derived from obstetric patients, while some studies included general surgical populations, potentially affecting PDPH risk profiles [20,26-28]. Most critically, a lack of standardized outcome definitions for PDPH (e.g., diagnostic criteria, severity scales, follow-up duration) makes direct comparison and synthesis challenging [21]. This heterogeneity explains why a clear, unified effect size is difficult to ascertain and underscores the need for standardized protocols in future research.

Interpretation in the Context of Existing Literature

Our narrative findings align with the broader, yet equivocal, conclusions found in previous meta-analyses. For instance, Deng et al. concluded that ITC placement significantly reduced both PDPH incidence and the need for therapeutic EBP [29], while Heesen et al., despite reporting similar risk reductions, emphasized through trial-sequential analysis that the evidence remains insufficient to conclusively exclude type I error [30]. This mirrors the tension within our own review: while the cumulative trend from cohort studies points toward benefit, the contradictory or null findings from RCTs and the high heterogeneity underscore the same methodological limitations and the need for more definitive trials [18-25]. Our analysis thus corroborates the existing literature's central dilemma: the intervention is promising but not yet proven.

Strengths and Limitations of the Included Studies

The included studies exhibit strengths, including diverse designs that provide a multifaceted view, some with large sample sizes enhancing generalizability, and a focus on a clinically relevant complication. However, limitations are significant: heterogeneity in design and outcomes complicates synthesis; retrospective and observational studies are prone to bias; small sample sizes in some studies limit power; and a focus on short-term outcomes leaves long-term sequelae unexplored.

Strengths and Limitations of the Systematic Review

This review adheres to PRISMA guidelines, employs a comprehensive multi-database search, and uses rigorous, independent screening and quality appraisal. Its primary limitation is the inability to perform a meta-analysis due to the heterogeneity of the included studies, which restricts quantitative synthesis. Furthermore, the review's scope is limited to short-term PDPH incidence, and the inclusion of non-English studies was not feasible.

Future Research Directions

Future research must prioritize large, pragmatic RCTs comparing ITC placement to standard management with standardized PDPH diagnostic criteria and follow-up. Studies should investigate optimal catheter management protocols (gauge, duration) and explore adjunct therapies. Longitudinal research is needed to assess long-term outcomes, including chronic headache risk.

## Conclusions

ITC placement appears to be a promising intervention for managing ADP, with much of the available evidence suggesting a reduction in PDPH incidence and a favorable safety profile. However, significant methodological limitations and heterogeneity within the evidence base prevent definitive conclusions. This review reinforces the necessity for further high-quality, standardized research to validate these findings and establish evidence-based clinical protocols.
